# Differences in perceived fairness and health outcomes in two injury compensation systems: a comparative study

**DOI:** 10.1186/s12889-016-3331-3

**Published:** 2016-07-29

**Authors:** Nieke A. Elbers, Alex Collie, Sheilah Hogg-Johnson, Katherine Lippel, Keri Lockwood, Ian D. Cameron

**Affiliations:** 1 John Walsh Centre for Rehabilitation Research, Sydney Medical School Northern, University of Sydney, Sydney, Australia; 2 Institute for Safety, Compensation and Recovery Research, Monash University, Melbourne, Australia; 3 Institute for Work and Health, Toronto, Toronto; 4 University of Ottawa, Ottawa, Canada

**Keywords:** Motor vehicle crash, Injury, Claimants, Procedural justice, Compensation systems

## Abstract

**Background:**

Involvement in a compensation process following a motor vehicle collision is consistently associated with worse health status but the reasons underlying this are unclear. Some compensation systems are hypothesised to be more stressful than others. In particular, fault-based compensation systems are considered to be more adversarial than no-fault systems and associated with poorer recovery. This study compares the perceived fairness and recovery of claimants in the fault-based compensation system in New South Wales (NSW) to the no-fault system in Victoria, Australia.

**Methods:**

One hundred eighty two participants were recruited via claims databases of the compensation system regulators in Victoria and NSW. Participants were > 18 years old and involved in a transport injury compensation process. The crash occurred 12 months (*n* = 95) or 24 months ago (*n* = 87). Perceived fairness about the compensation process was measured by items derived from a validated organisational justice questionnaire. Health outcome was measured by the initial question of the Short Form Health Survey.

**Results:**

In Victoria, 84 % of the participants considered the claims process fair, compared to 46 % of NSW participants (χ^2^ = 28.54; *p* < .001). Lawyer involvement and medical assessments were significantly associated with poorer perceived fairness. Overall perceived fairness was positively associated with health outcome after adjusting for demographic and injury variables (Adjusted Odds Ratio = 2.8, 95 % CI = 1.4 – 5.7, *p* = .004).

**Conclusion:**

The study shows large differences in perceived fairness between two different compensation systems and an association between fairness and health. These findings are politically important because compensation processes are designed to improve recovery. Lower perceived fairness in NSW may have been caused by potential adversarial aspects of the scheme, such as liability assessment, medical assessments, dealing with a third party for-profit insurance agency, or financial insecurity due to lump sum payments at settlement. This study should encourage an evidence informed discussion about how to reduce anti-therapeutic aspects in the compensation process in order to improve the injured person’s health.

## Background

Each year, in Australia, about 100,000 people acquire an injury after a motor vehicle crash [1]. An estimated 50 % of those lodge a compensation claim [2, 3]. They seek reimbursement for their damages, such as medical costs, loss of wages, and pain and suffering. Although the compensation process is supposed to improve recovery following injury, empirical literature indicates the contrary. People who lodge a compensation claim tend to show poorer recovery than those with similar injuries who do not claim [4–6]. The literature mainly distinguishes two explanations. The first is that injured claimants might not recover because there is a financial incentive not to get better as long as the claim process lasts (secondary gain) [7]. The second is that claimants have poorer recovery because of stress related to the compensation process. This is called secondary victimisation: first, people become a victim of the accident, then of the compensation system [8, 9].

The support for the latter theory comes from both quantitative and qualitative studies. According to these studies, compensation stress is caused by stressful interactions with insurers, many medico-legal assessments, limited and impersonal communication, lack of information, delayed reimbursement of medical costs and loss of income, being confronted with significant paperwork, and feelings of stigmatization and power imbalance between claimants and the other actors in the system [10–15]. Quantitative studies have not measured the claimant’s experiences of all these elements in one study, holistically. Furthermore, no study has been published yet comparing the experiences in different compensation systems. Measuring experiences of different claim aspects and comparing the experiences between jurisdictions is important, because it can give guidance about how to improve the compensation system.

The current study investigated the injured persons’ perceived fairness regarding all the claim elements in the compensation system together. The perceived fairness was compared in two systems, the scheme in New South Wales (NSW) and the scheme in Victoria, two neighbouring states in Australia. The main differences are: (i) The scheme in NSW is mainly fault-based which means that claimants have to prove that the other person was at-fault (the insurance company will perform a liability assessment to determine whether the other person was at-fault), whereas the scheme in Victoria is mainly no-fault, which means that claimants are compensated regardless of whether they or others were at-fault or not; (ii) In NSW medical assessments are relatively common and are conducted by a medical practitioner assigned by the insurance company, whereas in Victoria independent medical assessments are uncommon during the first 18 months of a claim; (iii) In NSW people receive a lump sum at settlement, including loss of income up to 100 % of previous earnings, whereas Victoria has intermittent payments every two weeks, including loss of income payments for those working at the time of injury, which generally replace 80 % of pre-accident earnings; (iv) In NSW, insurance is provided by seven private sector third-party insurers. Third-party insurance means that claimants deal with the insurance company of the person that caused the accident. The insurer in Victoria is the state government compensation authority, which is also the compensation system regulator and which recovers insurance premiums from all motor vehicles registered in the state, a sort of ‘first-party insurance’. More detailed information about both systems is provided in Table 1.

**Table 1:** Comparison of the Victorian and NSW compensation scheme design

	NSW motor accidents scheme	Victoria transport accident scheme
Scheme structure and administration		
1. Legislation	Motor Accidents Compensation Act	Transport Accident Act
2. What type of law governs the scheme: no-fault, hybrid, common law?	Hybrid. Mainly fault. Injured people have to prove that somebody else was at-fault. People can lodge a no-fault claim regardless of fault up to $5000. No-fault claims were excluded in this study.	Hybrid. Mainly no-fault. Injured people can claim regardless of fault. People with serious injury have access to common law, which is fault-based. Common law claims were excluded in this study.
3. Is the compensation scheme mechanism-based or disability-based?	Mechanism-based (injury resulting from motor vehicle/land-based transport accident)	
4. How does the compensation system interact with other societal structures?	Both systems purchase healthcare from national publicly funded and private healthcare systems. Both have involvement with legal systems for dispute resolution.	
5. Is the insurance compulsory? How is the scheme funded?	Compulsory insurance. Funded by annual insurance premiums paid by motor vehicle owners as part of registration.	
6. Does the jurisdiction insure through private carriers or a state insurance fund?	7 Third party private insurance companies (profit)	1 First party state government compensation agency (non-profit)
Scheme eligibility		
7. Is liability assessment a feature of the scheme?	Yes. Liability is assessed within 3 months after claim lodgement	No. Coverage is accepted for all transport accident related injuries.
8. What proportion of the total transport injury population is covered by the scheme? How is the total transport injury population defined?	The transport injury population are those who are injured in a transport accident that occurred in the state under investigation (respectively Victoria or NSW) AND anyone traveling in a vehicle registered by that particular state (respectively Victoria or NSW) in any part of Australia	
9. Which injuries and afflictions are covered and which are not? Are mental health claims covered?	All injuries arising from the transport accident are covered including mental injury.	
10. What is the time frame to lodge a claim?	The fault-based claim has to be lodged within 6 months post-injury	The no-fault claim has to be lodged within 12 months post-injury
Medical assessments		
11. Who conducts the medical assessments? What is the role of the physician in injury certification and fitness for work?	Medical assessments are conducted by doctors assigned by the insurance company or assigned by the injured person’s lawyer. For disputed medical assessments there is an independent medical assessment service	<18 months: assessments are conducted by injured person’s general practitioner. >18 months: there is a change in income replacement benefits so people still on benefits at this time commonly undergo a medical assessment by an assessor nominated by the TAC to determine work capacity
Scheme benefits and entitlements		
12. What benefits are paid for?	Compensation can be paid for medical and rehabilitation services, past (i.e. between injury and claim settlement) and future (i.e. after claim settlement) income replacement, travel, and household support, legal services, and pain and suffering	No-fault benefits include medical and rehabilitation services, income replacement, travel, and household support. Legal costs related to disputes (protocol disputes and Victorian Civil Appeals Tribunal) are reimbursed.
13. What is the level of income benefits/loss of wages? Are there caps on the wages earned?	Loss of wages involves 100 % of previous/future salary. Capped at $4,412 net weekly earning (2014). Payments are tax-free, provided certain conditions are met.	The claimant has to cover the first 5 working days before compensation of loss of earnings commences. <18 months: Income benefits will generally be 80 % of pre-accident weekly earnings, capped to a maximum of $1,250 per week (2014/15). Clients who were earning less than $612 will get 100 % of the pre-accident earnings. These payments are taxed. >18 months: Based on work capacity assessment. Payments are capped at $1,060 per week and they are not taxed. >36 months: Continuing benefits only with an impairment of at least 50 %
14. What is the duration and frequency of payments?	Treatment is paid as long as it is reasonable and necessary. Usually paid on an as incurred basis. Loss of income comprises 100 % of pre-injury earnings. Capped at $4,412 net weekly earning (2014). Loss of past and future income reimbursements are paid as a lump sum at claim settlement. Periodic financial hardship payment can be paid.	Treatment is paid as long as it is reasonable and necessary. Usually paid on an as incurred basis. Income benefits for no-fault claims are limited to 3 years from the accident unless they have a permanent impairment level of at least 50 %. Income benefits are paid fortnightly. Claims do not formally close.
Scheme Changes		
15. Has the compensation scheme undergone any significant changes during the study period?	No changes during the study period.	The claims process changed in October 2013. The main changes with respect to perceived fairness were an easier claims form and faster approval of services. Also a joint medical examination process was introduced reducing the number of medical assessments.

Table is based on information derived from the Transport Accident Act [33] and Motor Accidents Compensation Act [34] and checked by policy makers of both schemes. We used the format developed by Clay et al. [35] adapted for motor vehicle injury insurance schemes

The aim of the study was to compare the perceived fairness and health status of injured people claiming benefits through the compensation systems in NSW and Victoria. The no-fault compensation system in Victoria is hypothesised to be perceived as fairer and less stressful than in NSW, because the NSW system includes more adversarial components, such as the assessment of liability, medical assessments, lump sum payments and third-party insurance. It is also hypothesised that claimants in NSW would report poorer health outcomes than in Victoria due to these more adversarial components. To minimise potential cofounding, we adjusted for demographic characteristics, such as age, gender, socio-economic status and marital status.

## Method

### Participants

Participants were recruited via two large databases that hold personal injury claims information after a motor vehicle collision in Victoria and NSW. Using the rule of thumb to have 10–15 participants per predictor [16], we sought to recruit about 200 participants to allow for 15 independent variables in a regression model. Since we included two states, we aimed to recruit 100 participants per state.

In NSW, participants were selected from the claim database held by the Motor Accidents Authority (MAA). The MAA is the state government regulator of the seven third-party insurance companies dealing with personal injury compensation claims in NSW. Recruitment letters were sent to those who were over 18 years old, whose crash occurred 12 or 24 months ago, and whose claim was accepted. Only accepted claims were included because people whose claims are denied have only limited exposure to the compensation scheme. Equal numbers of people 12 and 24 months after injury were approached. Twelve and 24 months are common time points in compensation and health research [13, 17]. Each compensation system and each claim has different times at which things happen, so for the purpose of measuring the impact of the compensation system as a whole, general timeframes are chosen. Participants could opt-in by contacting the primary investigator [NE]. Those who had not replied were reminded at random, which continued until the required number of 100 participants was met. An experienced research nurse [KLo] conducted the interview by telephone. Verbal informed consent was obtained.

In Victoria, participants were selected from the claims database held by the Transport Accident Commission (TAC). The TAC is the state government-run first-party compensation agency dealing with compensation claims in Victoria. Recruitment letters were sent to people who were already enrolled in an on-going survey run by the TAC. The sample from the on-going TAC survey was considered representative for the Victorian claimant population. We sent letters to those who were over 18 years old, whose crash occurred 12 and 24 months ago, and whose claim was accepted. Equal numbers of people (12 and 24 months after injury) were approached. People could opt-out of the current study by contacting the Social Research Centre, which is an independent research organization that conducted the research on the TAC’s behalf. The Social Research Centre conducted the telephone interview. The research ethics committee of The University of Sydney approved the study.

### Questionnaire

The telephone interview involved a structured questionnaire consisting of seven categories: demographic variables, claim process, lawyer involvement, medical assessments, dispute resolution, health status, and work status.

### Demographic and injury factors

Demographic variables included age at time of the interview, gender, country of birth, postcode, highest education, and marital status. Socio-economic status (SES) was obtained by matching participants’ postcodes to a Census Collection District [18] (scale from 1 to 10; 1 = lowest SES, 10 = highest SES). Injury data was derived from the claims databases: (1) the type of injury, which is determined by trained insurance company personnel at the initial interview with the claimant, (2) number of days in the hospital. Time since the accident was either 12 or 24 months.

### Claim factors

Claim factors were lawyer involvement (yes/no), medical assessments assigned by the insurance/compensation agency (yes/no), and if yes, the number of assessments, dispute process (yes/no), claim status (NSW: yes/no, Victoria: claims do not close, so a proxy was used whether the claim was inactive for more than 6 months yes/no, which was derived from the claims database. Six months of inactivity is a timeframe used by the TAC, which is based on years of experience. A claim is considered inactive if there has not been any payment or correspondence between the TAC and the claimant for 6 months since the last payment/correspondence), previous claim (NSW: yes/no, Victoria: derived from the claims database), and whether the injured person was at fault or not (NSW: since they all have an accepted fault-based claim they have successfully proven that somebody else was at fault, so the NSW participants were indicated as being not-at-fault. It should be noted that claimants could have contributed to the harm they suffered through their own negligence, but they were still classified not-at-fault; Victoria: totally at fault, partially at fault, not at all at fault. We did not gather information as to whether someone else was at fault).

### Health and work status

Participants were asked to rate their general health pre-injury and their general health at the moment of the interview. Both were based on the first question of the SF12 [19], using a 5 point Likert scale: excellent to poor. Work status was determined by the question whether the participant was currently working part time or full time (yes/no). If not, they were asked whether they were unable to work permanently or temporarily or not working for some other health reason.

### Fairness perceptions

The fairness perceptions were clustered into 5 themes: claims process, claims management, medical assessments, lawyer involvement, and dispute resolution processes. Questions were mainly based on items of a validated organisational justice questionnaire [20], which has been applied to the compensation process in previous studies [17, 21]. Several questions were added through discussion with the TAC and MAA and based on the literature. Fairness questions were followed by a 5 point Likert answer scale (strongly disagree – strongly agree).

#### Claims process

To assess the general aspects of the claims process, participants were asked whether the amount of reimbursement they received so far was fair and acceptable (distributive justice). In addition, six self-developed questions were added to address other claim issues, such as whether it was difficult to fill in the forms, and whether the duration of the claim (so far) was acceptable.

#### Claims management

Participants were asked about the interaction with the claims manager: e.g. ‘the claims manager has taken into account my views and feelings during the claims management process’ (procedural justice), ‘the claims manager has provided me with the information I needed’ (informational justice), and ‘the claims manager treated me politely’ (interpersonal justice). Three questions were added about the approval of treatment requests and the promptness of approval of other services.

#### Lawyer involvement

In those cases in which the participants involved a lawyer, they were asked why a lawyer was needed (open-ended question), followed by three questions about the information provided by the lawyer (informational justice) and two questions about whether they received polite and respectful treatment (interpersonal justice). A final question concerned a self-developed item asking whether the lawyer made the claims process easier.

#### Medical assessments

If participants had attended an examination requested by the insurer/TAC, they were asked whether the number of medical assessments was acceptable. In addition, we asked whether the medical assessor had provided them with the information they needed, whether they carefully explained what was going to happen during the examination and whether the medical assessor examined them in an unbiased and objective way (informational justice). In addition, two questions addressed whether the medical assessor treated them politely and respectfully (interpersonal justice).

#### Dispute resolution

Those who had indicated that they were involved in a formal dispute resolution process were asked whether the decision maker in the dispute process provided them with the information needed, whether the way the judgement was made was explained carefully, and whether the decision maker had communicated the judgement to the participant (informational justice) and whether they received polite and respectful treatment (interpersonal justice). Finally, it was asked whether the dispute process was stressful. All questions were followed by a 5 point Likert answer scale. Multi-item scales were asked in a random order in Victoria as consistent with the usual practice of the research centre.

### Statistical analyses

In preparation of the analysis, several items were dichotomised: i.e. socio-economic status (low: 1–5 versus high: 6–10), education (low-medium versus high – high is defined as undergraduate, bachelor and postgraduate), marital status (married/de facto versus single/divorced/separated), type of injury (whiplash/soft tissue injury versus other), at-fault (totally versus not at all/partially), health (poor/fair versus good/very good/excellent) and all justice items (agree versus disagree/neutral). Type of injury was dichotomised into whiplash/soft tissue injury versus other type of injuries because whiplash is considered a problematic and very common injury in motor vehicle accident compensation systems [22].

Firstly, it was investigated whether participants in both samples had similar demographic characteristics. Secondly, we compared the fairness perceptions between NSW and Victoria. Pearson’s chi-square (for categorical variables) and t-tests (for continuous variables) were conducted. To check for selection bias, a non-response analysis was conducted comparing participants to those who received the recruitment letter but were not included. A NSW sub-sample analysis was conducted comparing whether the opt-in participants were different to those who were recruited through reminder phone calls.

Thirdly, it was investigated what factors are associated with the overall fairness perception. A multiple logistic regression analysis was conducted, with covariates added in two blocks, to model the probability that the process was considered fair (versus not fair/neutral). The first block included demographic and injury details; the second block added the claim factors. The claim factors were medical assessments, lawyer involvement, claim settlement, and previous claim. Dispute process (i.e. a formal internal appeal procedure, an external appeal procedure, or a civil trial in court) could not be included, because there were not enough participants involved in a dispute. ‘At-fault’ was not included because this variable only applies to the Victorian sample.

Finally, we explored whether the overall perceived fairness of the compensation process is associated with health status. Another multiple logistic regression analysis was conducted, with covariates added in two blocks, to model the probability of good or excellent health (versus fair or poor health). The first block explored the (unadjusted) association between the overall fairness perception and health. In the second block, demographic, injury variables, and health pre-injury are added.

IBM SPSS statistics software version 22 was used for all analyses.

## Results

### Participants

In NSW, 501 recruitment letters were sent out. In total, 25 participants opted in and 75 were included after a reminder phone call. After 100 participants were recruited, no further participants were sought. During the analysis, two participants were excluded because they were partners of injured people, they were not injured themselves, resulting in 98 NSW participants. Responders in NSW were significantly older than the non-responders, t (499) = 6.96, *p* < .001, but gender and time since accident were similar (χ^2^ = 3.78, *p* = .052; χ^2^ = 0.06, *p* = .805).

In Victoria, 149 participants were sent the information sheet, of which 103 participated. During the analysis, 19 were excluded because, after their data was linked with administrative data from the TAC, it was found that they had lodged a common law (i.e. fault-based) claim. Fault-based common law claims were excluded because the study design was to compare a fault-based system with a no-fault system. The fault-based common law claims in Victoria consist of a small category of severely injured people, experiencing a different system. This resulted in a total number of 84 Victorian participants. Non-response was somewhat higher in the < 25 year old group than in the > 45 year old group (42 % versus 25 %; only summarised frequency data was available so no test could be performed), but for the variable ‘time since injury’ the non-response was similar: about 30 % for both 12 months and 24 months after injury.

**Fig. 1:**
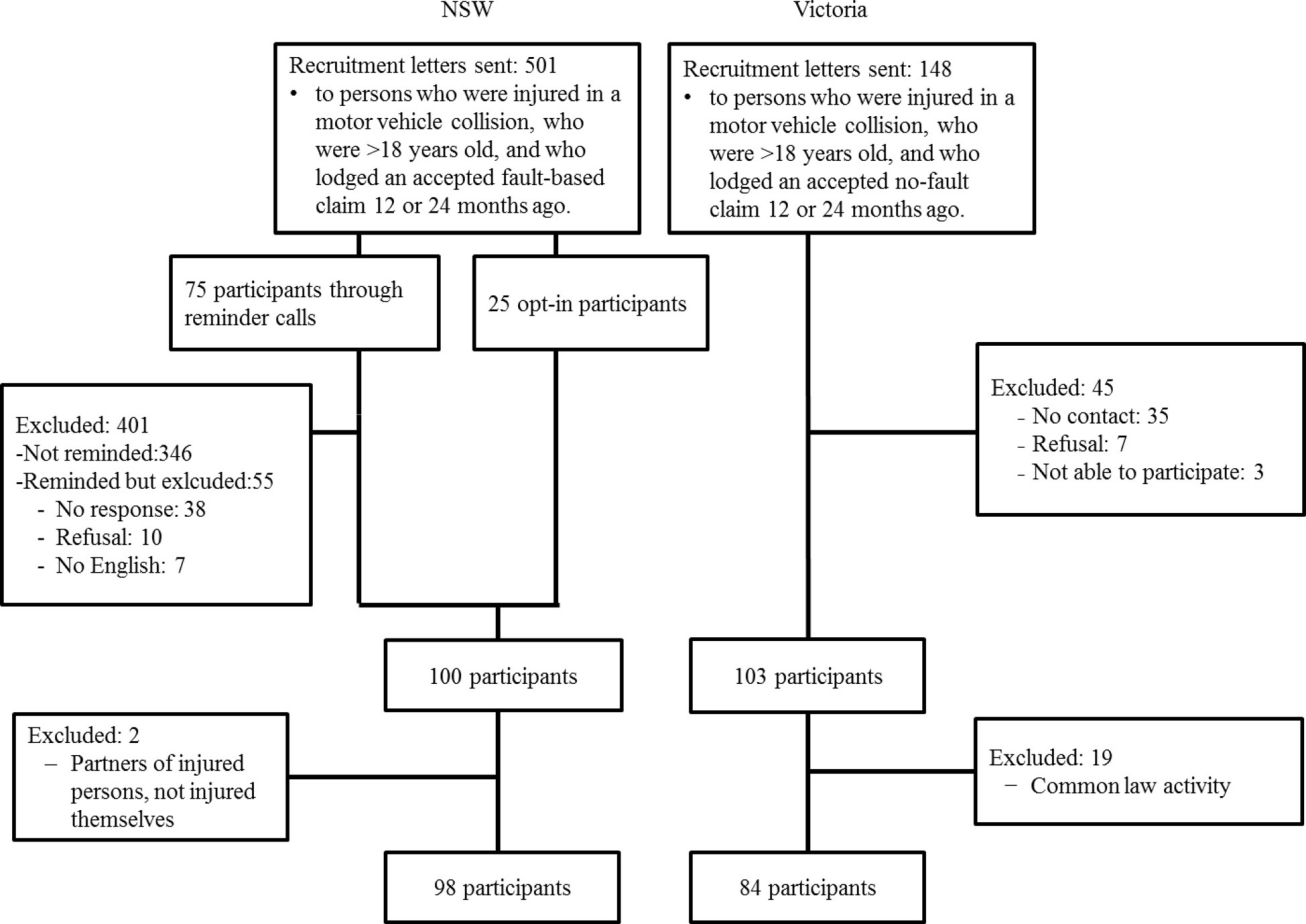
Flowchart of participants

The total number of participants in NSW and Victoria was 182. For 95 participants the injury occurred 12 months ago (NSW: 54; Victoria: 41), and 87 participants were included 24 months after injury (NSW: 44; Victoria: 43). The interviews were conducted from April to July 2014. It took on average 15 min to complete one interview. The flow chart of participants is provided in Fig. 1.

### Sample characteristics

The sample characteristics are displayed in Table 2.

**Table 2:** Sample characteristics, claim factors, health and work status

Demographic variables	NSW (*n* = 98)	VIC (*n* = 84)	χ^2^ or t (df)	*p*
	N (%), M (SD)	N (%), M (SD)		
Age	54.59 (14.36)	45.79 (16.27)	3.88 (180)	< .001
Gender (male)	52/98 (53 %)	58/84 (69 %)	4.84	.028
Country of birth (Australia)	67/98 (68 %)	61/84 (73 %)	0.39	.531
Socio-economic status (high)	46/98 (47 %)	51/84 (61 %)	3.45	.063
Education (high)	25/98 (26 %)	26/83 (31 %)	0.75	.386
Marital status (married)	54/98 (55 %)	44/82 (54 %)	0.04	.846
Injury (whiplash/soft tissue)	39/98 (40 %)	15/84 (18 %)	10.43	.001
Hospital (admitted)	43/98 (48 %)	54/84 (64 %)	7.57	.006
Time since the accident (12 months)	54/98 (55 %)	41/84 (49 %)	0.72	.397
Claim factors				
Lawyer	66/98 (67 %)	11/84 (13 %)	54.54	< .001
Medically assessed	53/98 (54 %)	8/83 (10 %)	39.73	< .001
Number of assessments	1.66 (1.02)	2.29 (1.38)	−1.47 (58)	.148
Dispute process	2/98 (2 %)	1/84 (1 %)	-	**-**
Claim status (settled/inactive)	25/98 (26 %)	38/84 (45 %)	7.78	.005
Previous claim	31/98 (32 %)	13/84 (16 %)	6.44	.011
Fault (at-fault)	0/98 (0 %)	14/79 (18 %)	18.86	< .001
Health and work status				
Health				
Pre-injury (good-excellent)	88/96 (92 %)	81/83 (98 %)	2.96	.085
Post-injury (good-excellent)	43/92 (47 %)	56/84 (67 %)	7.09	.008
Not working due to the accident^a^	12/98 (12 %)	15/84 (18 %)	1.13	.288

Notes. Socio-economic status (low = 1–5 versus high = 6–10), education (low-medium versus high; high is defined as undergraduate, bachelor and postgraduate), marital status (married/de facto versus single/divorced/separated), type of injury (whiplash/soft tissue injury versus other), at-fault (totally at fault versus not at all at fault/partially at fault), health (poor/fair versus good/very good/excellent)

^a^at time of interview

#### Demographic and injury variables

The NSW participants were older, more likely to be female, more likely to suffer whiplash injury, and less likely to have been admitted to the hospital. Country of birth, marital status, education, socio-economic status, and time since injury were similar in both states.

#### Claim factors

Claimants in NSW were more likely to have a lawyer than those in Victoria (67 % versus 13 %). The percentage of participants that was medically assessed by an assessor assigned by the insurance company was higher in NSW than in Victoria (54 % versus 10 %). For those who were assessed, the average number of assessments in NSW was similar to the average number in Victoria (two assessments on average). Three participants were involved in a dispute process: 2 in NSW and in 1 in VIC. In NSW, 26 % of claims were settled compared to 45 % inactive claims in Victoria. In NSW, 32 % had lodged a previous claim, compared to 16 % in Victoria. In NSW, all claimants were at least partially not-at-fault (they all had an accepted fault-based claim), whereas in Victoria 18 % considered themselves completely at fault.

#### Health and work

Pre-injury health status was similar between states. Post-injury, the participants in NSW had poorer health outcomes in comparison to participants in Victoria. The number of people not working due to the injury was not significantly different between states (NSW: 12 % vs Victoria: 18 %). A subgroup analysis of the NSW opt-in participants versus those who were recruited via reminder calls showed no differences in demographic, injury, claim, or health characteristics.

### Fairness perceptions

The fairness statistics are provided in Table 3.

**Table 3:** Fairness perceptions about the claims process, claims management, medical assessments, lawyer involvement, and dispute process

Claims process	NSW^a^	VIC^a^	χ^2^	*P*
It is easy to fill out forms	45/97 (46 %)	62/80 (78 %)	17.75	< .001
It is easy to support claim	50/97 (52 %)	70/82 (85 %)	23.00	< .001
Claim duration is acceptable	44/96 (46 %)	70/84 (83 %)	27.13	< .001
Compensation received so far is fair	45/98 (46 %)	60/81 (74 %)	14.50	< .001
Overall claim process is fair	44/96 (46 %)	70/83 (84 %)	28.54	< .001
Claims management				
The claims manager…				
takes views/feelings into account	39/96 (39 %)	62/82 (76 %)	22.05	< .001
manages claim objectively	41/96 (42 %)	78/84 (93 %)	50.29	< .001
uses correct information	43/96 (45 %)	75/82 (92 %)	43.11	< .001
provides information	44/96 (46 %)	71/83 (86 %)	30.56	< .001
explains procedure	33/96 (34 %)	64/79 (81 %)	38.15	< .001
communicates timely	39/95 (41 %)	66/84 (79 %)	25.88	< .001
is polite	66/96 (67 %)	78/82 (95 %)	19.90	< .001
is respectful	63/96 (64 %)	78/84 (93 %)	19.58	< .001
approves treatment needed	56/98 (57 %)	76/82 (93 %)	28.84	< .001
approves treatment promptly	51/98 (52 %)	68/82 (83 %)	19.01	< .001
approves other services promptly	37/98 (37 %)	49/73 (67 %)	14.43	< .001
Medical assessments				
The medical assessor…				
provided information	26/52 (50 %)	3/8 (38 %)	-	-
explained procedure	27/52 (52 %)	5/8 (63 %)	-	-
examined unbiased	21/52 (40 %)	3/8 (38 %)	-	-
was polite	35/52 (67 %)	6/8 (75 %)	-	-
was respectful	35/52 (67 %)	5/8 (63 %)	-	-
Number of assessments was acceptable	34/53 (64 %)	7/8 (88 %)	-	-
Lawyer involvement				
The lawyer…				
provided information	57/66 (86 %)	8/10 (80 %)	-	-
explained procedure	57/66 (86 %)	8/10 (80 %)	-	-
communicated timely	55/66 (83 %)	8/10 (90 %)	-	-
was polite	64/66 (97 %)	11/11 (100 %)	-	-
was respectful	61/66 (92 %)	11/11 (100 %)	-	-
The lawyer made the process easier	56/66 (85 %)	8/11 (73 %)	-	-
Dispute process				
Decision maker …				
provided information	0/2 (0 %)	1/1 (100 %)	-	-
explained procedure	0/2 (0 %)	1/1 (100 %)	-	-
communicated judgment	0/2 (0 %)	1/1 (100 %)	-	-
was polite	2/2 (100 %)	1/1 (100 %)	-	-
was respectful	2/2 (100 %)	1/1 (100 %)	-	-
Dispute process was stressful	2/2 (100 %)	1/1 (100 %)	-	-

Notes. The answer scale to all justice questions was strongly agree, agree, neither agree nor disagree, disagree, strongly disagree, which was dichotomised into strongly disagree, disagree, neither agree nor disagree versus agree, strongly agree

^a^column displays the number of participants that strongly agreed/agreed with the statement divided by the total number of participants that answered the question

- = The number of participants is too small to conduct further analyses

#### Claims process

In NSW, claimants consistently scored lower on all items of the claims process (*p* < .001). The overall fairness of the compensation process involved the largest difference (χ^2^ = 28.54), followed by the claim duration (χ^2^ = 27.13). Participants in NSW were less likely to agree that it was easy to provide the information needed to support the claim (χ^2^ = 23.00), or easy to fill in the forms (χ^2^ = 17.75), and they were less likely to consider the compensation amount received so far to be fair compared to Victorians (χ^2^ = 14.50). The percentage of participants who considered the different elements of the claims process to be fair ranged between 45 and 52 % in NSW, compared to 74 and 85 % in Victoria. The average fairness score of all claims process items was 3.06 (SD = 0.81) in NSW and 3.81 (SD = 0.90) in Victoria.

#### Claims management

NSW participants were more likely to report all actions of the claims manager to be less fair than participants in Victoria (*p* < .001). The largest difference was reported on the item whether the claims manager managed the claim objectively (χ^2^ = 50.29), followed by whether the claims manager used the correct information (χ^2^ = 43.11), whether he/she explained what was going to happen (χ^2^ = 38.15), and whether he/she provided the information that was needed (χ^2^ = 30.56). Timely communication was also valued quite differently between the two states (χ^2^ = 25.88). Whether the claims manager approved a treatment yielded a larger fairness gap between the two samples than whether the approval occurred promptly (resp. χ^2^ = 28.84 versus χ^2^ = 19.01). Whether the claims manager took the participant’s views and feelings into account (χ^2^ = 22.05), was polite (χ^2^ = 19.90), and respectful (χ^2^ = 19.58) resulted in a more modest difference. The percentage of participants perceiving the actions of the claims manager as fair ranged between 37 and 67 % in NSW versus between 67 and 95 % in Victoria. The average score for claims management by the insurance company in NSW was 3.19 (SD = 0.90) and 3.94 (SD = 0.68) in Victoria. In both states, the lowest score was for the approval of other services to be prompt (services such as travel expenses, home or gardening services, medical investigations) and highest score was for politeness. A subgroup analysis of the NSW opt-in participants versus those who were recruited via reminder calls showed no differences in perceived fairness regarding the claims process or the claims manager.

#### Medical assessments

In both states, not many participants agreed they were given information by the medical assessor (NSW: 50 %, Victoria: 38 %), nor were they likely to agree that the medical assessors explained what was going to happen (52 % in NSW; 63 % in Victoria). The lowest scores were acquired on the item whether the medical assessor was unbiased (40 % versus 38 %). In both states, the majority perceived being treated politely (NSW: 67 %; Victoria: 75 %) and respectfully (NSW: 67 %; Victoria: 63 %). Agreement with the actions of the medical assessor ranged between 40 and 67 % in NSW and 38 and 75 % in Victoria. The average score given to the interaction with the medical assessor was 3.18 (SD = 0.90) in NSW and 3.30 (SD = 1.06) in Victoria. The number of medical assessments was considered to be acceptable by more respondents in Victoria than in NSW (88 versus 64 %).

#### Lawyer involvement

In both states, participants were very satisfied with the information (NSW: 86 %; Victoria : 80 %) and the explanations that the lawyer provided (NSW: 86 %; Victoria : 80 %). The information was provided at the right time (NSW: 83 %; Victoria : 90 %). The lawyer was considered polite (NSW: 97 %; Victoria : 100 %) and respectful (NSW: 92 %; Victoria : 100 %). Agreement with the lawyers’ behaviour ranged between 83 %-97 % in NSW compared to 80 %-100 % in Victoria. The average score given to the interaction with their lawyer was 4.02 (SD = 0.49) in NSW and 3.80 (SD = 0.65) in Victoria. Participants indicated that the lawyer made the process easier (NSW: 85 %; Victoria: 73 %).

In both states, the main reason why participants involved a lawyer was because they believed they needed help/assistance/information in a process they were not familiar with and which they considered complicated (NSW: 36/66 = 55 %, Victoria: 5/11 = 45 %). Other reasons were that they were advised by others (NSW: 11/66 = 17 %, Victoria: 1/11 = 9 %), because they were not happy with a decision or outcome in the claims process (NSW: 7/66 = 11 %, Victoria: 2/11 = 18 %), or because the accident was not their fault/somebody else’s fault and they wanted to be sure they were rightfully compensated (NSW: 7/66 = 11 %, Victoria: 2/11 = 18 %).

#### Dispute process

Of the three participants who were involved in a dispute, the two participants in NSW were unhappy with the information, the explanations, and communication, whereas the one person from Victoria was satisfied. All three participants agreed that the decision maker was respectful and dignified, but they also all found the dispute process stressful.

### Predictors of overall fairness of the compensation process

The first model, in which only demographic and injury factors were included, showed that married participants were more likely to perceive the compensation process as fair than singles (Adjusted Odds Ratios [AOR] = 2.11, *p* = .027). Furthermore, people with whiplash/soft tissue injuries were less likely to perceive the compensation process as fair than those with other types of injuries (AOR = 0.43, *p* = .035). The second model, in which the claim factors were included, showed that being medically assessed (AOR = 0.31; *p* = .013) and having a lawyer involved (AOR = 0.33; *p* = .016) were independently negatively associated with overall fairness of the compensation process. Marital status and injury type were no longer significant. There was no multicollinearity. The AOR’s and p-values are shown in Table 4.

**Table 4:** Predictors overall fairness of the compensation process

Independent variables	Overall fairness claims process							
	Model 1				Model 2			
	AOR	CI		*p*	AOR	CI		*p*
Age	0.99	0.97,	1.01	.21	1.00	0.97,	1.02	.73
Gender	0.75	0.38,	1.48	.41	0.79	0.37,	1.68	.54
Country of birth	0.91	0.44,	1.87	.79	0.87	0.38,	1.97	.73
Socio-economic status	1.84	0.95,	3.56	.07	1.38	0.65,	2.94	.41
Education	0.58	0.28,	1.21	.15	0.46	0.20,	1.06	.07
Marital status	2.11	1.09,	4.06	.03	1.69	0.81,	3.55	.16
Injury	0.43	0.19,	0.94	.04	0.65	0.26,	1.60	.35
Hospital admission	0.73	0.36,	1.49	.38	1.08	0.48,	2.45	.85
Time after injury	1.04	0.54,	1.99	.91	1.08	0.48,	2.44	.86
Medical assessment					0.31	0.12,	0.78	.01
Lawyer involvement					0.33	0.13,	0.81	.02
Claim status					1.71	0.68,	4.32	.26
Previous claim					1.66	0.68,	4.03	.26

Notes: Model 1 Nagelkerke R^2^ = .111; Model 2 Nagelkerke R^2^ = .339

Multiple logistic regression analysis, modelling the probability that the process was considered fair (versus not fair/neutral). The first model includes demographic and injury details. The second model adds the claim factors. ‘At-fault’ was not included because this variable only applies to the Victorian sample. There was no multicollinearity

Coding: Gender (0 = Male; 1 = Female); Country of birth (0 = Other; 1 = Australia); Socio-economic status (0 = Lower; 1 = Higher); Education (0 = Low/Medium; 1 = High); Marital status (0 = Single/Divorced; 1 = Married); Injury (0 = Other; 1 = Whiplash/soft tissue injury); Hospital admission (0 = No; 1 = Yes); Time after injury (0 = 12 months; 1 = 24 months). Medical assessment (0 = No; 1 = Yes); Lawyer involvement (0 = No; 1 = Yes); Claim status (0 = Pending; 1 = Settled); Previous claim (0 = No; 1 = Yes); Overall fairness claims process (0 = not fair/neutral, 1 = fair). Reference category = 0

### Predictors of health post-injury

The first model, which investigated the unadjusted association, showed a significant positive interaction between overall fairness perception of the compensation process and health post-injury (OR = 2.78, *p* = .002). The second model, which adjusted for demographic and injury characteristics, showed that the association between fairness and health was of the same magnitude after adjustment (AOR = 2.83, *p* = .004). Additionally, health pre-injury was associated with health post-injury (AOR = 6.15, *p* = .039). There was no multicollinearity. The AOR’s and *p*-values are shown in Table 5.

**Table 5:** Predictors of health post-injury

	Health post-injury							
	Model 1				Model 2			
Independent variable	OR	CI		*p*	AOR	CI		*p*
Fairness claims process	2.78	1.45,	5.33	.002	2.83	1.40,	5.71	.004
Age					1.01	0.99,	1.04	.23
Gender					0.91	0.45,	1.83	.78
Country of birth					1.00	0.48,	2.12	.99
Socio-economic status					1.12	0.57,	2.20	.74
Education					0.88	0.42,	1.88	.75
Marital status					0.90	0.46,	1.79	.77
Injury					1.35	0.57,	3.16	.49
Hospital admission					2.04	0.97,	4.26	.06
Time after injury					0.62	0.32,	1.20	.16
Health pre-injury					6.15	1.09,	34.61	.04

Note: Model 1 Nagelkerke R^2^ = 0.08; Model 2 Nagelkerke R^2^ = 0.16

Multiple logistic regression analysis, modelling the probability of good or excellent health (versus fair or poor health). Model 1 explores the unadjusted association between the overall fairness perception and health. Model 2 adjusts for demographic, injury variables, and pre-injury health. There was no multicollinearity

Coding: Overall fairness claims process (0 = disagree/neutral; 1 = agree); Gender (0 = Male; 1 = Female); Country of birth (0 = Other; 1 = Australia); Socio-economic status (0 = Lower; 1 = Higher); Education (0 = Low/Medium; 1 = High); Marital status (0 = Single/Divorced; 1 = Married); Injury (0 = Other; 1 = Whiplash/soft tissue injury); Hospital admission (0 = No; 1 = Yes); Time after injury (0 = 12 months; 1 = 24 months); Health pre-injury (0 = Poor; 1 = Good); Health post-injury (0 = poor/fair, 1 = good/excellent). Reference category = 0

## Discussion

The study investigated whether there were differences in perceived fairness between two injury compensation schemes of different design. The findings reported here suggest the answer to this question is yes. Based on the raw fairness perceptions, participants in the fault-based, lump-sum payment system in NSW considered the compensation process less fair than the participants in the no-fault, intermittent payment system in Victoria. The lower fairness perceptions concerned the claims process in general (e.g. claim lodgement, duration of the process, and the amount of compensation), how the claims manager dealt with the claim (e.g. objectiveness, prompt approval of treatment / other services) and how the claims manager interacted with the claimant (e.g. providing information, timely communication, being respectful). In contrast, participants in both states who had hired a lawyer were equally very satisfied with their lawyer, which indicates that NSW participants are not more negative overall. Participants in both states perceived the interaction with the medical assessor as being reasonably fair. The participants in NSW reported lower post-injury health status than in Victoria (note that these are raw comparisons without adjusting for sample differences).

The study also sought to determine whether differences in perceived fairness were associated with health status post injury. The analyses show that, after adjustment for demographic and injury characteristics, the overall perceived fairness of the compensation process was positively associated with the injured person’s health. Those who perceived the overall compensation process to be fair were almost three times more likely to report good health outcomes. The association between perceived fairness and health recovery is consistent with what is shown in two previous studies [17, 23].

The elements negatively contributing to the overall fairness perception of the compensation process were lawyer involvement and medical assessments. The association between lawyer involvement and lower perceived fairness could be multidirectional. One possible direction is that lawyers could contribute to the perception of unfairness. Once a lawyer is involved, the compensation agency generally will ensure all requests for information or advice are sent directly to the claimant’s legal representative [24], which means that the injured person’s experiences depend on the information provided by the lawyer. However, lawyer involvement could also be the result of perception of unfairness and complexity of the scheme [25]. In the current study, the majority of participants involved a lawyer to assist with navigation of the compensation process, and participants indicated that the lawyer made the process easier, which seems to support the latter explanation, although the first one cannot be ruled out.

The association between medical assessments and lower perceived fairness could be explained by the fact that people who are medically assessed may consider that the purpose of assessment is to challenge the validity of their claim [12, 26]. Our findings suggest that the medical assessment may be associated with lower perceived fairness because the medical assessor arranged by the insurer was considered to be biased.

Besides lawyer involvement and medical assessments, there are four system design differences that could explain the fairness difference. Firstly, the compensation systems differ in fault/no-fault nature. In NSW the insurance companies have to assess whether their client (i.e. the insured driver who was involved in the collision) was responsible/at-fault/liable for the crash whereas in Victoria compensation is provided regardless of fault. Liability assessment has been identified as a potential source of stress [13], as it can take up to 3 months to be decided, and therefore causes insecurity of and delay in compensation payments. It should be noted that we only included participants with accepted claims. Including participants whose claim was not accepted because liability was denied, could have resulted in a higher rate of perceived unfairness regarding the claim lodgement in NSW [13].

Secondly, in NSW in 100 % of cases, someone else, aside from the participant was at least partially at-fault, whereas in Victoria 18 % of participants indicated that they were at-fault. A subgroup analysis of the Victorian sample showed that those who were at-fault considered the process to be fairer than those who were not at all/partially at fault (t (76) = − 3.0, *p* = .004; not reported in the result section), which is in line with a previous study [27]. Those who perceive themselves to have been at fault for their own injury could perceive the process to be fairer, being grateful to receive compensation despite the fact that they were at fault. Those whose injury is attributable to someone else’s fault may want acknowledgment of the harm that has been inflicted. This need for acknowledgement is not always met which could lead to perceived unfairness [28].

Thirdly, Victoria has one state government compensation agency, whereas in NSW seven for-profit third-party insurance companies are in place. For-profit insurers have a stronger financial incentive to minimise the costs of compensation which may influence their actions. Injured persons might have less trust in for-profit, third-party insurance companies which could lead to poorer perceived fairness. The literature has shown that trust in authorities moderates procedural justice [29]. Further, there is likely to be a greater diversity of practices within the multiple insurance companies in NSW that influences perceived fairness, whereas in Victoria there is a single claims management agency with presumably less diversity in process.

The fourth difference is the frequency of compensation payment, in general, and the payment of loss of income, more specifically. In Victoria, loss of income is paid every two weeks to those who were working at the time of accident, whereas in NSW loss of income is paid as a lump sum at the settlement of the claim. As the average minor fault-based compensation claim takes two years to settle (personal communication with MAA), waiting that long for loss of income payments to be paid (at settlement) can create a significant financial burden [11, 30, 31] and therefore be likely to contribute to lower fairness perceptions in NSW, among those who have not returned to work. Participants in Victoria were more likely to consider the compensation received so far to be acceptable than those in NSW. The chi square statistic was not as large as for the other items, possibly because only 12 % of the NSW subsample indicated to be work disabled at the time of the interview.

A limitation of the current study is a possible selection bias, showing from the sample differences. For practical reasons, a different recruitment method was used in NSW versus Victoria (opt-in versus opt-out, participants were approached by a single person versus a research centre, and the research centre randomised the order of multi-item scales). Some differences (e.g. age, gender) could be due to the different recruitment method, others (e.g. in medical assessments, lawyer involvement) represent the actual circumstances in the compensation systems. It should also be noted that the findings may not be generalizable to other states or countries with either a fault or a no-fault system, because there are differences *within* fault-based systems and no-fault systems. A fault or no-fault system can be pure or modified with add-on no-fault or fault-based elements [22, 32]. Both NSW and Victoria have modified systems. As we selected only fault-based claims in NSW, the current findings may not be generalizable to all claimants in NSW. Similarly, the findings may not be generalizable to all claimants in Victoria, because we excluded common law (i.e. fault-based) claims. It should also be noted that systems in other countries operate in other social contexts, including variations in health care and other social benefits available, and that there may be other attitudes towards injury and social rights [22]. However, the elements discussed in this paper are present in all systems, and the aim is to generate a discussion about what design is anti-therapeutic and what we can do to improve the fairness experience. Finally, it is a cross sectional study, thus temporality cannot be assessed.

The research findings lead to potential suggestions for improvements. For example, applying first-party insurance instead of third-party insurance could increase perceived fairness, as the insurance company may be more committed to the injured person being a client. Furthermore, both the claims manager and the medical assessor could pay more attention to providing information before and after the medical assessment. A proposal to overcome potential bias in medical assessments is to have an independent organisation to broker the provision of examiners [26]. In addition, it seems better to provide interim payments rather than paying a lump sum at settlement, as this will provide financial security throughout the process, which is needed for the injured person to focus on recovery. It seems likely that these improvements will lead to improved perceived fairness. Improved perceived fairness may subsequently result in less lawyer involvement, which in turn may lead to greater well-being.

## Conclusion

This study shows that there are differences in perceived fairness reported by injured people who lodge a compensation claim in two different compensation systems in Australia. The differences in fairness and in systems, together with the finding that perceived fairness is associated with the injured persons’ health, suggests that the design of a compensation system can have a detrimental effect on the injured person’s health and recovery.

An association between perceptions of unfairness and poorer recovery in the compensation process is concerning and politically important because compensation processes are designed to *improve* recovery. Although further research is required to validate and generalise the findings, the study could act as a catalyst to an evidence-based discussion on how to reduce anti-therapeutic aspects of the compensation process in order to improve the injured person’s health and recovery after a motor vehicle crash.

## Abbreviations

AOR, adjusted odds ratio; MAA, Motor Accidents Authority; NSW, New South Wales; SES, socio-economic status; TAC, transport accident commission
